# MELAS Missed for Years: Stroke-Like Lesions Are No Indication for Brain Biopsy

**DOI:** 10.1155/2019/9312451

**Published:** 2019-12-27

**Authors:** J. Finsterer

**Affiliations:** Krankenanstalt Rudolfstiftung, Messerli Institute, Vienna, Austria

## Abstract

A 56-year-old female with a history of chronic alcoholism until age 38 y with a relapse between ages 45 and 46 y developed seizures, psychosis, and hemianopia to the left at age 46 y. Imaging revealed a right parieto-occipital lesion with intralesional bleeding. Five months after the first lesion she developed a second left parieto-occipital lesion, resulting in cortical blindness. Extensive workup, including brain biopsy, was noninformative. Retrospectively, the occipital abnormalities were identified as stroke-like lesions (SLLs). Further manifestations of the mitochondrial disorder (MID) were tremor, cerebral atrophy, bilateral basal ganglia, calcification, glaucoma, hypoacusis, short stature, hyperostosis frontalis, hyperthyroidism, sick-sinus syndrome and AV-block-1, and myopathy. According to the Walker criteria, a possible MID was diagnosed. In conclusion, adult-onset MID may be missed for years, SLLs may be easily misinterpreted entailing brain biopsy, and psychosis may contribute to a reduced impact for proper workup of a MID.

## 1. Introduction

Despite recent advances in the management of mitochondrial disorders (MIDs), diagnosing and treating MIDs remains challenging [1]. This is not only due to unawareness of the condition in adults but frequently also due to misinterpretation of diagnostic tests and ignorance. Therapy is challenging as well, as no causal treatment is currently available for most of the conditions [2], but progress has been achieved in preventing transmission of MIDs via the maternal line (maternal spindle transfer and pronuclear transfer) [3]. Here, we present a patient with a late-onset, multisystem MID, which remained undiagnosed for 10 y.

## 2. Case Report

**Figure 1:**
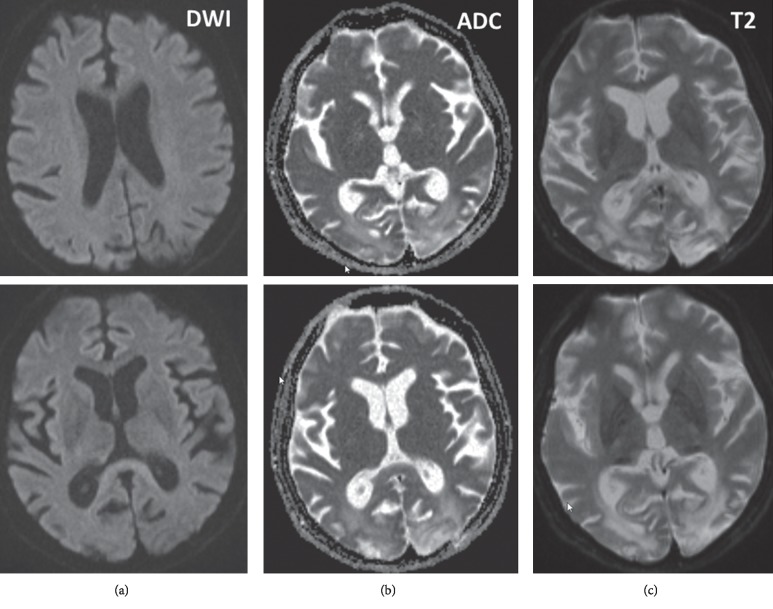
Cerebral MRI, 2 months after onset of psychosis, epilepsy, and visual impairment showing a slightly hyperintense right occipital lesion and a slightly hypointense similar lesion in the left occipital area, which were both hyperintense on ADC and hyperintense on T2-weighted images. Within the right occipital lesion, cortical and subcortical bleeding was identified. The occipital lesions were retrospectively interpreted as consecutive, subacute SLLs.

The patient is a 56-year-old Caucasian female, with height 168 cm and weight 57 kg, who was referred for replacing carbamazepine (CBZ) by another antiepileptic drug (AED), which does not cause hyponatremia or thrombocytopenia. She had a long-term medical history suggestive of a slowly progressive multisystem disease (Table 1), including fracture of the petrosal bone after a collapse of unknown cause at age 16 y, right bimalleolar fracture at age 23 y, chronic alcoholism until age 38 y with a relapse between ages 45 and 46 y, and icterus and hepatopathy at age 39 y. At age 43 y she received bilateral breast implants. At age 46 y, she developed epilepsy initially with a series of partial complex and generalized seizures, being attributed to chronic alcoholism, but the latter occurred during abstinence. Additionally, she experienced a first psychotic episode with optic hallucinations and developed hemianopia to the left. Workup with imaging initially revealed a right occipital lesion hyperintens on diffusion-weighted imaging (DWI), apparent diffusion coefficient (ADC), and T2-weighted images with bleeding in the center of the lesion in the absence of arterial hypertension or coagulopathy and a small lesion on the left precentral gyrus. Five months later, a left occipital lesion (hyperintense on DWI, ADC, and T2-weighted images) not confined to a vascular territory developed (Figure 1). Workup by means of imaging, functional studies, cerebrospinal fluid (CSF) investigations, blood tests, electroencephalography (EEG), and brain biopsy via a left occipital drill-hole, for primary cerebral neoplasm, metastasis, extrapontine myelinolysis, hepatic encephalopathy, Macchia-Fava disease, neuro-Whipple disease, the Heidenhain type of Creutzfeldt-Jakob disease, cerebral vasculitis, including neuro-Behcet, Wernicke-encephalopathy, and dementia, was negative. Thus, the occipital lesions were interpreted as posterior reversible encephalopathy syndrome (PRES). The lesions were followed up during the succeeding years, evolving into gliosis, cysts, and siderosis and retrospectively interpreted as stroke-like lesions (SLLs). Cortical blindness, psychosis, and epilepsy persisted.

At age 47 y, transient arterial hypertension without requiring long-term medication and hyponatremia, first recognized after adding oxcarbazepine (OXC), became apparent. OXC was replaced with CBZ. An AV-block-1 was recorded. At age 49 y, a pacemaker was implanted because of recurrent syncopes due to sinus arrest. Additionally, bilateral glaucoma was diagnosed. At age 53 y, ischemic stroke in the left posterior cerebral artery territory occurred. She reported muscle cramps of the small foot muscles (carpopedal spasms) and hypoacusis since years (Table 1). The patient had various AEDs since onset of epilepsy, including levetiracetam (LEV), OXC, LTG, lacosamide (LAC), and CBZ, but adherence to these drugs was poor. The family history was positive for breast cancer in her mother. Since age 53 y, the index patient was supported by a legal representative.

Clinical neurologic exam at age 56 y revealed short stature, hypoacusis, bilateral ptosis, marked bilateral visual impairment , left-sided intention tremor, wasting of the distal lower limb muscles, and reduced Achilles tendon reflexes bilaterally. Creatine kinase (CK) and resting serum lactate were normal. There was mild hyponatremia and thrombocytopenia being attributed to CBZ and mild hepatopathy. Though the CBZ serum level was within the normal range, CBZ was replaced by LEV (1500 mg/d). ECG showed an AV-block-I. Gastroscopy revealed a Barrett esophagus, and why esomeprazole was given. EEG showed occasional spikes in the right temporal projection. Cerebral computed tomography (CT) showed generalized atrophy and the known left temporal and bilateral occipito-temporal defects. The mitochondrial multiorgan disorder syndrome (MIMODS) score was 46 (number of organs affected: 6, number of organ manifestations: 15: PubMed hits: 25) (Table 2) [4]. Possible MID was diagnosed [4]. The patient refused to undergo further diagnostic workup for MID and announced not to take the AEDs prescribed.

## 3. Discussion

The presented patient is interesting for slowly progressive multimorbidity with onset at age 46 y, suggesting a MID and matching with the diagnostic criteria for possible MID [5, 6]. Arguments for the diagnosis of MID are seizures, tremor, psychosis, cerebral atrophy, bilateral basal ganglia calcification, and the bilateral SLLs, suggesting cerebral involvement, glaucoma suggesting ophthalmologic involvement, hypoacusis suggesting involvement of the ears, short stature, hyperostosis frontalis, and hyperthyroidism suggesting endocrine involvement, sick-sinus syndrome and AV-block-1 suggesting cardiac involvement, and bilateral ptosis, distal lower limb wasting, and carpopedal spasms, suggesting myopathy. A further argument for MID is the MIMODS score of 46. MIMODS scores >10 have been shown to be indicative for a MID [4]. A late-onset MID is likely also in the light of an increasing number of mtDNA mutations with advanced age [7].

According to a recent consensus statement, a SLE can be diagnosed solely upon clinical presentation, cerebral MRI, and EEG [personal communication]. No advance genetic confirmation is required. The reason why the SLE had been misinterpreted and the SLL missed on MRI remains elusive. Most likely, a SLL was not considered as a differential and only those mentioned above were excluded. Arguments for a SLE are the MRI findings, the clinical presentation, the disease trajectory, and the multisystem nature of the condition. An argument against the right occipital SLL could be the bleeding since SLLs usually do not go along with intracerebral bleeding, but it is conceivable that in the course of laminar cortical necrosis, bleeding and consecutive siderosis developed. A further argument for a SLL is the dynamics of the lesions ending up as atrophy, gliosis, cysts, and siderosis. Arguments against PRES in the presented patient are that the occipital lesions did not resolve (PRES last maximally for three months), that arterial hypertension was well controlled even without antihypertensive drugs, that epilepsy and psychosis are only rare manifestations of PRES [8, 9], and that cortical blindness did not resolve. An argument against a reversible cerebral vasoconstriction syndrome is that the lesion lasted >3 months.

To which degree the right occipital stroke at age 53 y further deteriorated visual acuity remains speculative as the patient could not specify if visual acuity further deteriorated after this cerebrovascular event. Whether the 10 y history of psychosis contributed to the delayed suspicion and diagnosis of a MID remains speculative, but it is conceivable that psychiatric disease may generally prevent from a thorough workup for neurological disease as in nonpsychiatric patients with similar complaints [10]. Limitations of the study were that first-degree relatives were not systematically investigated and that muscle biopsy and genetic workup were refused by the patient and her legal representative.

## 4. Conclusions

This case shows that late-onset MIDs may be missed for years, that SLLs may be easily misinterpreted even entailing brain biopsy, and that the history of psychosis may contribute to a reduced impact for a proper workup of an underlying MID.

## Figures and Tables

**Table 1:** Disease trajectory of the presented patient.

Onset, age	Manifestation	Therapy
16 y	Collapse with TBI (petrous bone fracture)	Conservative
23 y	Right bimalleolar fracture	Np
Until 38 y	Chronic alcoholism	Withdrawal
39 y	Icterus, hepatopathy	None
43 y	Small breasts	Breast silicon implants
45–46 y	Relapse of alcoholism	Successful withdrawal
46 y	Epilepsy with focal and generalized seizures	Levetiracetam
46 y	1 SLL with intralesional bleeding (right occipital) hemianopia and neglect to the left, psychosis	Neuroleptics
46 y	Hepatopathy, steatosis hepatis	None
Until age 46 y	Smoking	Withdrawal
46 y	Acne rosacea	Topic ointments
46 y	Arachnoidal cyst (left temporo-basal)	None
46 y	Folic acid deficiency	Folic acid
46 y	Hyperlipidemia	Diet
46 y	2 SLE (left occipital (epilepsy, psychosis, cortical blindness, and optic hallucinations))	Brain biopsy via drill-hole quetiapin
47 y	Arterial hypertension	Bisoprolol
47 y	AV-block-1	None
47 y	Hyponatremia after adding OXC	OXC replaced with CBZ
Since age 47 y	Thrombocytopenia after adding CBZ	CBZ withdrawal
47 y	Myoclonic seizure	Levetiracetam
49 y	Glaucoma	Latanoprost
Until 49 y	Recurrent syncopes due to sinus arrests	Pacemaker
49 y	Hyperthyroidism	None
53 y	Capsular fibrosis	Replacement of breast implants
53 y	Ischemic strokes (PCAS territory, PCAS stenosis)	Antithrombotics
56 y	Barrett esophagus	Esomeprazole
56 y	Hyperostosis frontalis	None
56 y	Generalized brain atrophy	None
Since years	Carpopedal spasms	Magnesium
Since years	Hypoacusis	None

PCAS: left posterior cerebral artery syndrome, PRES: posterior reversible encephalopathy syndrome, SLE: stroke-like episode, AEDs: antiepileptic drugs, SSS: sick-sinus syndrome, TBI: traumatic brain injury, CBZ: carbamazepine, and np: not provided.

**Table 2:** MIMODS score calculated as the sum of three items.

(1) Number of affected organs (range: 0–14)
(muscle, CNS, endocrine organs, heart, intestines, nerves, ears, eyes, bone marrow, kidneys, skin, bones, lungs, and arteries)
(2) Number of manifestations per organ
*Muscle* (ophthalmoplegia (3), ptosis (3), easy fatigability (exercise intolerance) (3), limb weakness (2), hyper-CK-emia (2), wasting (1), respiratory insufficiency (1), myoglobinuria (1), cramping (1), myalgia (1), abnormal lactate stress test (1), double vision (diplopia) (1), muscle stiffness (1), fasciculations (1), muscle rupture (1))
*CNS* (epilepsy (3), atrophy (3), ataxia (3), Parkinson (3), dementia (3), stroke-like episodes (2), migraine (2), dystonia (1), tremor (1), leukoencephalopathy (white matter lesions) (1), spasticity (1), dysarthria (1), psychosis (1), confusion (1), mild cognitive impairment (1), basal ganglia calcification (1), dilative arteriopathy (1), delayed visually evoked potentials (1), myelopathy (1))
*Heart* (hypertrophic, dilated, restrictive CMP (3), heart failure (2), systolic dysfunction (2), arrhythmias (1), arterial hypertension (1), noncompaction (1), pulmonary hypertension (1), aortic root ectasia (1), coronary heart disease (1), congenital heart disease (1))
*Intestines* (hepatopathy (3), vomiting (1), diarrhea(1), pancreatitis (1), steatosis hepatis (1), nonspecific colitis (1), diverticulosis (1), sialadenitis (1))
*Nerves* (neuropathy (3), neuronopathy (1))
*Eyes* (optic atrophy (3), retinopathy (3), cataract (1), glaucoma (1))
*Ears* (hypoacusis (3), tinnitus (1))
*Kidneys* (renal insufficiency (2), renal failure (2), tubular acidosis (1), Fanconi syndrome (1), cysts (1), nephrolithiasis (1))
*Arteries* (dissection (1), spontaneous rupture (1), aneurysm (1), ectasia (1), dilative arteriopathy (1), atherosclerosis (1))
*Bone marrow* (anemia (2), leukopenia (1), thrombopenia (1))
*Bones* (skeletal deformities (1), polyarthralgia (1), arthrosis (1))
*Dermis* (psoriasis (1), lipomatosis (1), vitiligo (1), edema (1), hypertrichosis (1), baldness, alopecia (1), madarosis (1), skin rashes (1), seborrheic eczema (1)^*∗*^)
(3) Frequency of citations in PubMed (>100 citations: 3 points, 50–100 citations: 2 points, <50 citations; 1 point); ^*∗*^appropriate scores are provided in parenthesis above

A score >11 suggests definite MID; ^*∗*^the PubMed value is provided in parenthesis after each manifestation.
